# What constitutes a "clinical trial"?: A survey of oncology professionals

**DOI:** 10.1186/1745-6215-9-12

**Published:** 2008-03-03

**Authors:** James R Wright, Brenda Kowaleski, Jonathan Sussman

**Affiliations:** 1 Department of Oncology, McMaster University, Hamilton, Ontario, Canada; 2 Juravinski Cancer Centre at Hamilton Health Sciences, Hamilton, Ontario, Canada

## Abstract

**Background:**

What constitutes a "clinical trial" is inconsistently defined in the medical literature. With an initiative by Cancer Care Ontario (CCO) to report institutional clinical trials activity across the province of Ontario, Canada, we sought to investigate the variability in the interpretation of the term by local oncology professionals.

**Methods:**

A survey amongst the physicians and nurses at the Juravinski Cancer Centre at Hamilton Health Sciences, Ontario was conducted. The survey included 12 summaries of local clinical research studies, and respondents were asked which they believed represented a clinical trial. Subsequently, they were asked which of the same 12 studies they believed should be labeled as clinical trials when considering separate definitions provided by CCO and by the Ontario Cancer Research Network (OCRN).

**Results:**

A total of 66 (54%) of 123 surveys were completed; 32/46 (70%) by physicians, 21/59 (36%) by primary care nurses, and 13/18 (72%) by clinical trial nurses. Without a standardized definition, all studies, 12/12, were considered to be clinical trials by at least 50% of respondents. When provided with the CCO definition only 6/12 studies were considered to be clinical trials by the majority of respondents, while with the OCRN definition it was 9/12 studies. Studies evaluating natural health products, non-traditional medical interventions, and non-randomized studies with standard interventions consistently ranked the lowest, regardless of the definition used.

**Conclusion:**

Oncology professionals appear to have a broadly inclusive baseline definition of what constitutes a clinical trial. Establishing rigor and consistency in the definition of a clinical trial is important for any program, institutional or jurisdictional based comparisons of clinical trials activity, especially when used as a quality indicator of patient care.

## Background

Clinical trials are appreciated as the most important vehicle by which advances in patient management occur [1]. Unfortunately only a small number of cancer patients participate in clinical trials [2-5]. This has lead to various national and regional strategies to try and improve overall trials activity. Summary measures to evaluate this activity within institutions or across jurisdictions have typically been reported as the proportion of new patients, either within an institution or from population based incidence statistics, or the number of treated patients, that are enrolled in clinical trials [6,7]. This proportion of patients is suggested to represent an institutional measure of quality of care, with the hypothesis that a higher proportion of patients enrolled in clinical trials is reflective of higher quality of care [8]. However, a number of assumptions underlie the interpretation of such a simple summary measure, including the ability to consistently and reliably define the types of clinical research studies that should be designated as "clinical trials".

Cancer Care Ontario (CCO) monitors the performance of the cancer system in the Province of Ontario, Canada. As part of this monitoring CCO has recently proposed twenty-five "quality indicators" as summary measures of the Ontario cancer system, all of which are available to the general public [8]. One of these quality measures is the proportion of treated cancer patients enrolled in clinical trials. Data is available from all 12 of the regional cancer programs across the province. The CCO website presents their operational definition of a clinical trial as being "treatment based; including surgery, systemic chemotherapy and radiation therapy. Trials for cancer prevention, screening or diagnosis are not included". CCO obtains this data regarding trials activity from the Ontario Cancer Research Network (OCRN), which is a not-for-profit, provincially funded organization, whose key mandate is to increase clinical trials activity in the province of Ontario. Many cancer programs have received infrastructure-funding support from the OCRN to increase patient accrual to clinical trials, with the intention of doubling baseline enrollment levels. The OCRN's definition of a clinical trial as contained within the OCRN Clinical Trials Infrastructure Fund Policy Manual, suggests that a clinical trial is one testing a new therapy and quotes a US based National Institutes of Health (NIH) definition as "a research study to answer specific questions about vaccines or new therapies or new ways of using known treatments."

Given the potential for variably interpreting the above definitions of a clinical trial, we sought to investigate the types of clinical research that our local oncology professionals considered to be clinical trials, and how they interpreted the CCO and OCRN definitions in comparison.

## Methods

A single institution survey was circulated by internal mail to all physicians and nurses at the Juravinski Cancer Centre (JCC). The JCC is home to a large comprehensive regional cancer program within Hamilton Health Sciences, Hamilton, Ontario, Canada. The centre serves a regional population of approximately 2.3 million people. Most physicians and nurses at the JCC are responsible for the care of patients involved in clinical research studies. After receiving approval from our local Research Ethics Board (REB), the survey was sent to a total of 123 individuals; consisting of 46 physicians and 77 nurses of whom 18 nurses were from the clinical trials department. A covering letter explaining the survey was included (see Additional file 1). Individual survey responses were tracked solely for the purpose of a mailed reminder to complete the survey. A second copy was sent to non-responders after a 6-week period.

The survey was constructed from brief descriptions of 12 previously or currently active local clinical research protocols, all of which required local REB approval and signed informed consent from the involved patients. The 12 studies were purposely selected to encompass the breath of the active research portfolio, and to ensure various phases and intensity of research were represented. The study summaries were initially reviewed with two nurses and two physicians to ensure clarity and comprehensiveness. The 12 studies selected included the following (with question number in brackets, see Additional file 1):

• 2 studies evaluating new drugs or vaccines, one phase I (Q6), the other a randomized phase III (Q11)

• 2 randomized studies comparing standard therapies, both phase III (Q1, Q8)

• 2 studies prospectively evaluating natural products, both phase II (Q2, Q9)

• 2 randomized studies evaluating medical interventions that are not direct cancer therapies (i.e. not surgery, radiation, or systemic chemotherapy), both phase III (Q10, Q12)

• 2 studies prospectively evaluating non-traditional medical interventions that are not cancer therapies, one phase II (Q5), the other a randomized phase III (Q4)

• 2 non-randomized studies with standard interventions prospectively correlating outcome measures (Q3, Q7)

Without providing a definition, respondents were asked to indicate which of the 12 research summaries they believed to be a "clinical trial". Response options included yes, no, or unknown. Respondents were then provided with the CCO and the OCRN definitions as quoted previously, and were again asked to assess each research summary using the definition provided. The covering page stressed the need to complete the survey in this prescribed order. This resulted in a total of 36 separate responses; each study assessed without a definition; each study using the CCO definition; and each study using the OCRN definition. A full copy of the survey is available (see Additional file 1).

### Analysis

The age, gender, and profession of all respondents were collected. Percentages were used to represent the overall proportion of affirmative responses for each of the 12 studies, using each of the three definitions. The proportion of respondents that changed a response within a question triplet was recorded. The proportion of affirmative responses for each set of 12 questions, by professional group, was compared with Chi-squared and Fisher's Exact Tests to determine statistically significant differences. Exploratory analysis with t-tests and analysis of variance were performed within and between the professional groups.

## Results

A total of 66 of the 123 (54%) surveys were returned, 32 from physicians (32/46 = 70%), 21 from primary care nurses (21/59 = 36%), and 13 from clinical trial nurses (13/18 = 72%). Overall the 32 physicians represented 49% of the respondents, and included 13 medical oncologists, 11 radiation oncologists, 5 surgical oncologists, and 3 general practitioners in oncology. The primary care nurses represented 32% of respondents, and clinical trial nurses 20%. Fifty-one, or 77% of respondents were between the ages of 35 and 55 years of age.

The percentages of affirmative responses for each question, and the mean score per definition, are summarized in Table 1. When respondents utilized their personal definition of what constituted a clinical trial, all questions were answered affirmatively by at least 50%, noting that question 3, dealing with tumor markers, was exactly 50%. Four other questions, specifically numbers 5, 7, 4, and 9, in rank order of affirmative responses, had scores ranging between 61% and 73% respectively.

**Table 1:** Affirmative Response Rates by Study by Each Definition (n = 66)

**Definition**	**Personal (Q1–12)**	**CCO (Q13–24)**	**OCRN (Q25–36)**
Questions			
Q1, 13, 25	97%	94%	92%
Q2, 14, 26	86%	44%	71%
Q3, 15, 27	50%	49%	50%
Q4, 16, 28	67%	29%	35%
Q5, 17, 29	61%	21%	38%
Q6, 18, 30	89%	88%	96%
Q7, 19, 31	64%	41%	44%
Q8, 20, 32	89%	83%	88%
Q9, 21, 33	73%	49%	70%
Q10, 22, 34	89%	62%	80%
Q11, 23, 35	97%	97%	99%
Q12, 24, 36	92%	73%	83%
Mean	80%	61%	68%

When provided with the CCO definition of a clinical trial, only half the studies were considered to represent a clinical trial. Questions 5, 4, 7, 2, 3 and 9 as ranked in order of their percent affirmative responses, were all below 50%. While with the OCRN definition, nine of the 12 studies were scored by more than 50% to represent a clinical trial. Questions 4, 5, and 7 ranked below 50% with scores of 35%, 38%, and 44% respectively. When the summary of each research project was considered in the context of all three definitions, i.e. questions 1, 13, 25 and questions 2, 14, 26 etc each as a question triplet there were five questions (2, 4, 5, 7, 9) that had 50% or more of respondents change at least one affirmative response based on the definition in use (see Table 2). For all 12 of the question triplets, on average just over one third of respondents changed at least one response. Regardless of the definition utilized, questions 2, 3, 4, 5, 7, and 9 consistently ranked lowest for affirmative response scores.

**Table 2:** Proportion of Respondents (n = 66) that Changed at Least one Response per Question Triplet

**Question Triplets**	**% Changed Responses**
Q1, 13, 25	14%
Q2, 14, 26	56%
Q3, 15, 27	39%
Q4, 16, 28	62%
Q5, 17, 29	56%
Q6, 18, 30	17%
Q7, 19, 31	56%
Q8, 20, 32	21%
Q9, 21, 33	50%
Q10, 22, 34	36%
Q11, 23, 35	3%
Q12, 24, 36	27%
Mean	36.5%

Across groups, physicians and clinical trial nurses were generally similar in their level of affirmative responses. The primary care nurses tended to be more conservative with fewer affirmative responses. Using their personal definition of a clinical trial, they scored significantly lower than both physicians and trial nurses, or physicians alone for questions 2, 4, 5, 6, and 8 (see Table 3). Analysis of variance for profession, gender and age, found only professional group was significant for the rates of positive responses. Analysis within the three definitions separately found that profession predicted differences only with the personal and CCO definitions, but not the OCRN definition (only data for the personal definition is presented in Table 3). Within the physician and nursing groups there was no gender or age effects noted.

**Table 3:** Affirmative Response Rates for Personal Definitions by Study by Professional Groups

	**Physician (n = 32)**	**Clinical Trial Nurse (n = 13)**	**Other Nurses (n = 21)**
Questions			
Q1	100%	100%	91%
Q2	100%	100%	57%*
Q3	47%	69%	43%
Q4	84%	62%	43%**
Q5	72%	69%	38%**
Q6	100%	100%	67%*
Q7	63%	69%	62%
Q8	100%	92%	71%**
Q9	81%	77%	57%
Q10	94%	92%	81%
Q11	100%	100%	91%
Q12	97%	100%	81%
Mean	87%	86%	65%

* Significantly lower than both physicians and clinical trial nurses (p < 0.05)

** Significantly lower than physicians alone (p < 0.05)

## Discussion

This survey illustrates a number of important issues. First, that there is variability in what oncology professionals personally define as being a clinical trial. Nine of the 12 research summaries had less than 90% affirmative responses, and four had between 50% and 67% with affirmative responses, suggesting at least some degree of collective uncertainty. Secondly, simple nuances in the definition of a clinical trial can have a major impact on the interpretation of whether the described research study constitutes a clinical trial or not. In this survey, on average, over one third of respondents changed at least one of their responses for each study based on the applied definition. Perhaps not surprisingly, given the focus on treatment based clinical trials, the operationalized CCO definition as provided to respondents was the most strictly interpreted, as only 6/12 studies were considered to represent clinical trials by the majority of respondents, vs. 9/12 with the OCRN definition, and 12/12 without a standardized definition. Thirdly, as the preceding ratios demonstrate, most oncology professionals have a personal definition of a clinical trial that is broadly inclusive of most clinical research.

The research studies that consistently ranked the lowest in all situations included studies evaluating natural products (questions 2 and 9), non-traditional medical interventions (questions 4 and 5), and studies with standard interventions and no prospective comparison groups (questions 3 and 7). In part this likely reflects a bias towards the traditional medical model of investigational therapeutics.

This variability interpreting what constitutes a clinical trial is not surprising. At one extreme, a strict interpretation of the Health Canada or the Food and Drug Administration (FDA) regulations is that clinical trials are research studies evaluating new drugs, or vaccines, or new indications for such existing agents. In Canada, only these types of research studies require a "No Objection Letter" from Health Canada after a Clinical Trial Application (CTA) outlining the proposed research is submitted and reviewed. Research studies comparing standard therapies, or approved agents such as radiation therapy, regardless of the dose, do not require a similar level of federal review. But this narrow pharmacologically based interpretation does not appear to be widely utilized. In a recent editorial outlining the rational for the registration of future clinical trials, the International Committee of Medical Journal Editors have defined a clinical trial as "any research project that prospectively assigns human subjects to intervention and comparison groups to study the cause-and-effect relationship between a medical intervention and a health outcome" [9]. While an intervention in this context is broadly defined to include drugs, surgical procedures, devices, behavioral treatments, process-of-care changes and the like, the definition is clearly limited to randomized phase III trials. Others such as Pocock, have suggested that a clinical trial is "any form of a planned experiment which involves patients and is designed to elucidate the most appropriate treatment of future patients with a given medical condition", specifically including classic phase I and II trials within this definition [10].

In oncology, various definitions and classification schemes for clinical trials exist. The US National Cancer Institute (NCI) website suggests, "clinical trials are research studies in which people help doctors find ways to improve health and cancer care" [11]. The NCI classifies clinical trials as treatment trials, prevention trials, screening trials, diagnostic trials, genetics trials, and quality of life trials. Quality of life trials, also referred to as supportive care trials, are described as trials "to reduce side effects from primary treatments, other symptoms, and the beneficial effects of nutrition, group therapy and other approaches". The NCI website suggests that population and family-based genetic research studies are not cancer clinical trials as traditionally defined. In distinction however, the ClinicalTrials.gov website, supported as a service of the US NIH more broadly defines a clinical trial as a research study in human volunteers to answer specific health questions, and categorizes trials as interventional, or observational. Observational trials are defined as trials that address health issues in large groups of people or populations in natural settings [12]. This definition would suggest that studies involving tumour banking, without a planned intervention per se would be considered as clinical trials if they follow a pre-defined protocol. Some ambiguity is even reflected in the web sites of specific cancer treatment Programs. At MD Anderson for example, clinical trials are defined as "strictly controlled human studies of new and emerging therapies". Yet, the largest treatment category listed under active clinical trials on their website is "no treatment" [13].

This survey was limited in scope and complexity. The purposeful sampling of research projects accentuates the true variation that would result from nuances of a definition of a clinical trial, and the total scores per definition have little inherent meaning. Respondents, while discouraged from doing so, had the opportunity to read through the entire survey prior to completing the sections in the prescribed order. Such activity however, would have likely resulted in less, not more variation in their responses. Despite a second mailing the survey was only completed by 54% of the solicited physicians and nurses. From a phenomenistic perspective, many of the primary care nurses informally noted concern with the apparent complexity of the survey and the perception of a large time requirement. A well-accepted trials definition may have facilitated more timely completion. Physicians and clinical trial nurses are most directly involved in clinical research, and were the groups most likely to complete the questionnaire, and perhaps as a bias of their positions they also tended to report a higher proportion of affirmative responses. Within these two groups the response rate was a more compelling 70 plus percent. A larger survey would seem unlikely to either result in different conclusions or remove the need to develop and utilize a robust definition of what constitutes a "clinical trial".

## Conclusion

The intent of this survey was not to develop a definition of what constitutes a clinical trial, but rather to illustrate the importance of doing so. If oncology professionals within the same institution are not consistent with their interpretation of what constitutes a clinical trial it would seem unlikely that health care administrators or the public at large would have a meaningful appreciation of the issues. It is not always clear how investigators or clinical research programs have defined a clinical trial when reporting their rates of clinical trials activity. A meaningful and consistently applied definition of a clinical trial is important to establish as programmatic and institutional activity summaries are generated across the province of Ontario and beyond. Such comparisons may facilitate a broader understanding of the recruitment process. Even local trends in clinical trials recruitment must be examined with a consistently applied definition if the recruitment process is to be better understood and improved. Before interpreting any recent improvements in patient recruitment, it is important to ensure that changes are not simply a function of a more inclusive definition of a clinical trial. A consistent definition of a clinical trial is not an issue unique to oncology, as all investigators with an interest in improving the metrics of patient participation in clinical research need to be speaking the same language.

## Abbreviations

CCO: Cancer Care Ontario; CTA: Clinical Trial Application; FDA: Food and Drug Administration; JCC: Juravinski Cancer Centre; NCI: National Cancer Institute; NIH: National Institutes of Health; OCRN: Ontario Cancer Research Network; REB: Research Ethics Board.

## Competing interests

The authors declare that they have no competing interests.

## Authors' contributions

JW and BK conceived of the survey. JW, BK, and JS developed the survey, reviewed the data and drafted and reviewed the manuscript. All authors read and approved the final manuscript.

## Supplementary Material

Additional file 1 Defining a clinical trial in oncology: Information sheet and survey. This is a copy of the information sheet, the definitions and the survey circulated to medical professionals. Click here for file

## References

[B1] Sackett D, Haynes B, Guyatt G, Tugwell P (1991). Clinical Epidemiology: A Basic Science For Clinical Medicine (ed2).

[B2] Cassileth BR (2003). Clinical Trials: Time for Action. J Clin Oncol.

[B3] Wittes RE, Friedman MA (1988). Accrual to Clinical Trials. J Natl Cancer Inst.

[B4] American Medical Association Council on Scientific Affairs (1991). Viability of Cancer Clinical Research: Patient accrual, coverage, and reimbursement. J Natl Cancer Inst.

[B5] Winn RJ (1994). Obstacles to the Accrual of Patients to Clinical Trials in the Community Setting. Seminars in Oncol.

[B6] Corrie P, Shaw J, Harris R (2003). Rate limiting factors in recruitment of patients to clinical tirals in cancer research: descriptive study. BMJ.

[B7] Murthy VH, Krumholz HM, Gross CP (2004). Participation in Cancer Clinical Trials. JAMA.

[B8] Cancer Care Ontario Website. http://www.cancercare.on.ca/qualityindex2007/evidence/trials/index.html.

[B9] (2005). Is This Clinical Trial Fully Registered? – A Statement from the International Committee of Medical Journal Editors. NEJM.

[B10] Pocock S (1994). Clinical Trials: A Practical Approach.

[B11] National Cancer Institute Website. http://www.cancer.gov/clinicaltrials/learning/what-is-a-clinical-trial.

[B12] ClinicalTrials.gov Website. http://www.clinicaltrials.gov/ct/info/whatis#whatis.

[B13] MD Anderson Cancer Center Website. http://www.mdanderson.org/patients_public/clinical_trials/display.cfm?id=7A230A82-7513-11D4-AEBD00508BDCCE3A&method=displayFull.

