# Supplementary material for: What constitutes a "clinical trial"?: A survey of oncology professionals
Source: Trials. 2008 Mar 3;9:12. doi: 10.1186/1745-6215-9-12 (PMC2267771; doi:10.1186/1745-6215-9-12)
Supplement: Additional file 1 — Defining a clinical trial in oncology: Information sheet and survey. This is a copy of the information sheet, the definitions and the survey circulated to medical professionals. [file 1745-6215-9-12-S1.doc]

Appendix I.

Defining a Clinical Trial in Oncology.

Information Sheet and Survey

We are conducting a survey to better understand what health care professionals mean when they say a research study is a “Clinical Trial”.

We are asking physicians and nurses to review the following information and to consider completing the attached questionnaire; it should take approximately 10-12 minutes to complete.

If you are agreeable, please answer each of the sections in the order they appear; i.e. do the first 12 questions in Section I, then the second 12 questions in Section II, and then the last 12 questions in that order.

Your completion of the survey will be considered as implicit consent.

We will track the ID numbers on each survey only for the purpose of reminding you of this survey in approximately 2 and 4 weeks time. If you do not wish to complete the survey please return it with this clearly written across the first page, to ensure you receive no reminders. Once the survey is complete, no personal opinions will be available at any time; only the summarized data will ever be presented. Results of the survey will be available to all respondents.

To thank you for your time, if you have any questions or concerns please contact us through the clinical trials department at extension 64417.

Jim Wright and Brenda Kowaleski

Version Date: July 25, 2005

Defining a Clinical Trial in Oncology

Clinical Trials lead to advances in patient management. The proportion of patients that participate in clinical trials is low, with commonly quoted estimates in the range of 3-5%.

The proportion of patients in clinical trials has been suggested as an institutional measure of quality of care, with the assumption that the more patients enrolled in clinical trials the better. A single summary statistic, such as the proportion of new patients or treated patients that are enrolled in clinical trials, has obvious appeal.

But such a summary measure is associated with a number of assumptions, the most obvious being the ability to define a clinical trial.

The following is a list of local research projects, each of which had a full protocol, required and obtained full REB approval, and required patients to sign an informed consent form.

Please read each study description and indicate whether you feel it represents a clinical trial.

#### SECTION I

**With your current understanding of what defines a clinical trial, please indicate which of the following 12 studies you would consider to be a “clinical trial”?**

1. A study in which patients with early stage breast cancer were randomized to receive 3 weeks of radiation or 5 weeks of radiation to the breast. The primary outcome was the incidence of breast cancer recurrence.

YES NO UNSURE

1. A study in which patients with advanced non-curative cancer were randomized to receive a food supplement with omega-3 oils or a food supplement without. The primary outcome was the degree of weight gain.

YES NO UNSURE

1. A study in which patients with lung cancer that had received high dose radiation therapy with or without chemotherapy, had levels of various proteins measured from tumor biopsies, the results of which were then correlated with the tumor response to radiation therapy.

YES NO UNSURE

1. A study in which women with node positive breast cancer were randomized to the use a decision board, or not, as an aid to decide whether or not to receive adjuvant chemotherapy. The primary outcome was their decisional conflict score.

YES NO UNSURE

1. A study in which patients that had completed adjuvant chemotherapy for breast cancer were entered into an exercise program. The primary outcome was weight loss, and the amounts of weight gain or loss were compared to that of an historical cohort.

YES NO UNSURE

1. A study in which patients with advanced non-curative solid tumors were treated with increasing doses of a modified viral agent to determine the dose at which patients began to experience severe but temporary side effects.

YES NO UNSURE

1. A study in which patients with head and neck cancer that were to receive high dose radiation therapy, had weekly assessments of the lining of their mouth (mucosa) and blood work performed to determine if levels of C-reactive protein in the blood correlated with the visible degree of mucosal damage.

YES NO UNSURE

1. A study in which patients with head and neck cancer that were to receive high dose radiation treatment to the oral cavity were randomized to either morning or afternoon radiation therapy. The primary outcome was the incidence of higher-grade mucositis.

YES NO UNSURE

1. A study in which patients with colon cancer that were to be treated with a specific type of chemotherapy likely to cause diarrhea were provided a natural food product containing high doses of a specific amino acid. The primary outcome was the reduction in the frequency of bowel movements, when compared to historical controls.

YES NO UNSURE

1. A study in which patients with head and neck cancers that were expected to experience dry mouth symptoms following radiation therapy were randomized to nerve stimulation (codetron) or not during radiation treatment. The primary outcome of the trial was the measured amount of timed salivary flow 4 weeks following completion of radiation therapy.

YES NO UNSURE

1. A study in which patients with advanced stage lung cancer were randomized to the use of a new oral EGFR drug or placebo, after completing standard chemo-radiotherapy. The primary outcome was the overall survival of patients in the two groups.

YES NO UNSURE

1. A study in which patients with advanced or metastatic lung cancer were randomized to 12 weeks of weekly Epoetin alpha or placebo injections. The primary outcome of the trial was the difference in the change in quality of life scores from baseline to 12 weeks.

YES NO UNSURE

**SECTION II (Please ensure section I is complete before proceeding)**

Cancer Care Ontario defines a clinical trial as being “treatment based, including surgery, systemic therapy and radiation therapy. Trials for cancer prevention, screening or diagnosis are not included”

**Thinking about this CCO definition of a clinical trial, please indicate which of the following same 12 studies you would consider to be a clinical trial.**

1. A study in which patients with early stage breast cancer were randomized to receive 3 weeks of radiation or 5 weeks of radiation to the breast. The primary outcome was the incidence of breast cancer recurrence.

YES NO UNSURE

1. A study in which patients with advanced non-curative cancer were randomized to receive a food supplement with omega-3 oils or a food supplement without. The primary outcome was the degree of weight gain.

YES NO UNSURE

1. A study in which patients with lung cancer that had received high dose radiation therapy with or without chemotherapy, had levels of various proteins measured from tumor biopsies, the results of which were then correlated with the tumor response to radiation therapy.

YES NO UNSURE

1. A study in which women with node positive breast cancer were randomized to the use a decision board, or not, as an aid to decide whether or not to receive adjuvant chemotherapy. The primary outcome was their decisional conflict score.

YES NO UNSURE

1. A study in which patients that had completed adjuvant chemotherapy for breast cancer were entered into an exercise program. The primary outcome was weight loss, and the amounts of weight gain or loss were compared to that of an historical cohort.

YES NO UNSURE

1. A study in which patients with advanced non-curative solid tumors were treated with increasing doses of a modified viral agent to determine the dose at which patients began to experience severe but temporary side effects.

YES NO UNSURE

1. A study in which patients with head and neck cancer that were to receive high dose radiation therapy, had weekly assessments of the lining of their mouth (mucosa) and blood work performed to determine if levels of C-reactive protein in the blood correlated with the visible degree of mucosal damage.

YES NO UNSURE

1. A study in which patients with head and neck cancer that were to receive high dose radiation treatment to the oral cavity were randomized to either morning or afternoon radiation therapy. The primary outcome was the incidence of higher-grade mucositis.

YES NO UNSURE

1. A study in which patients with colon cancer that were to be treated with a specific type of chemotherapy likely to cause diarrhea were provided a natural food product containing high doses of a specific amino acid. The primary outcome was the reduction in the frequency of bowel movements, when compared to historical controls.

YES NO UNSURE

1. A study in which patients with head and neck cancers that were expected to experience dry mouth symptoms following radiation therapy were randomized to nerve stimulation (codetron) or not during radiation treatment. The primary outcome of the trial was the measured amount of timed salivary flow 4 weeks following completion of radiation therapy.

YES NO UNSURE

1. A study in which patients with advanced stage lung cancer were randomized to the use of a new oral EGFR drug or placebo, after completing standard chemo-radiotherapy. The primary outcome was the overall survival of patients in the two groups.

YES NO UNSURE

1. A study in which patients with advanced or metastatic lung cancer were randomized to 12 weeks of weekly Epoetin alpha or placebo injections. The primary outcome of the trial was the difference in the change in quality of life scores from baseline to 12 weeks.

YES NO UNSURE

**SECTION III (please ensure the first two sections are completed before proceeding)**

The Ontario Cancer Research Network (OCRN) defines a clinical trial as one testing a new therapy and quotes an National Institutes of Health (NIH) definition as “a research study to answer specific questions about vaccines or new therapies or new ways of using known treatments.”

**Thinking about this OCRN-NIH derived definition of a clinical trial, please indicate which of the following same 12 studies you would consider to be a clinical trial.**

1. A study in which patients with early stage breast cancer were randomized to receive 3 weeks of radiation or 5 weeks of radiation to the breast. The primary outcome was the incidence of breast cancer recurrence.

YES NO UNSURE

1. A study in which patients with advanced non-curative cancer were randomized to receive a food supplement with omega-3 oils or a food supplement without. The primary outcome was the degree of weight gain.

YES NO UNSURE

1. A study in which patients with lung cancer that had received high dose radiation therapy with or without chemotherapy, had levels of various proteins measured from tumor biopsies, the results of which were correlated with the tumor response to radiation therapy.

YES NO UNSURE

1. A study in which women with node positive breast cancer were randomized to the use a decision board, or not, as an aid to decide whether or not to receive adjuvant chemotherapy. The primary outcome was their decisional conflict score.

YES NO UNSURE

1. A study in which patients that had completed adjuvant chemotherapy for breast cancer were entered into an exercise program. The primary outcome was weight loss, and the amounts of weight gain or loss were compared to that of an historical cohort.

YES NO UNSURE

1. A study in which patients with advanced non-curative solid tumors were treated with increasing doses of a modified viral agent to determine the dose at which patients began to experience severe but temporary side effects.

YES NO UNSURE

1. A study in which patients with head and neck cancer that were to receive high dose radiation therapy, had weekly assessments of the lining of their mouth (mucosa) and blood work performed to determine if levels of C-reactive protein in the blood correlated with the visible degree of mucosal damage.

YES NO UNSURE

1. A study in which patients with head and neck cancer that were to receive high dose radiation treatment to the oral cavity were randomized to either morning or afternoon radiation therapy. The primary outcome was the incidence of higher-grade mucositis.

YES NO UNSURE

1. A study in which patients with colon cancer that were to be treated with a specific type of chemotherapy likely to cause diarrhea were provided a natural food product containing high doses of a specific amino. The primary outcome was the reduction in the frequency of bowel movements, when compared to historical controls.

YES NO UNSURE

1. A study in which patients with head and neck cancers that were expected to experience dry mouth symptoms following radiation therapy were randomized to nerve stimulation (codetron) or not during radiation treatment. The primary outcome of the trial was the measured amount of timed salivary flow 4 weeks following completion of radiation therapy.

YES NO UNSURE

1. A study in which patients with advanced stage lung cancer were randomized to the use of a new oral EGFR drug or placebo, after completing standard chemo-radiotherapy. The primary outcome was the overall survival of patients in the two groups.

YES NO UNSURE

1. A study in which patients with advanced or metastatic lung cancer were randomized to 12 weeks of weekly Epoetin alpha or placebo injections. The primary outcome of the trial was the difference in the change in quality of life scores from baseline to 12 weeks.

YES NO UNSURE

Thank you…..please use the attached envelope to mail us back your responses.
